# Association between maternal hepatitis B virus carrier and gestational diabetes mellitus: a retrospective cohort analysis

**DOI:** 10.1186/s12985-021-01691-0

**Published:** 2021-11-20

**Authors:** Wanchang Yin, Bingjun Chen, Yilin Yang, Xiuzi Li, Ruirui Li, Jiangnan Xie, Guixian Chen, Fang He, Dunjin Chen

**Affiliations:** 1 Department of Obstetrics and Gynecology, The Third Affiliated Hospital of Guangzhou Medical University, Guangzhou, China; 2 Key Laboratory for Major Obstetric Diseases of Guangdong Province, Guangzhou, China

**Keywords:** Hepatitis B surface antigen, Deoxyribonucleic acid load, Gestational diabetes mellitus

## Abstract

**Introduction:**

Given that many pregnant women have chronic hepatitis B virus (HBV) infection and that gestational diabetes mellitus (GDM) is linked to poor maternal and neonatal outcomes, we looked into the relationship between the hepatitis B surface antigen (HBsAg) and GDM to see if a high HBV DNA load is linked to a higher risk of GDM in chronic maternal HBsAg carriers.

**Materials and methods:**

Our study included 39,539 pregnant women who gave birth at the Third Affiliated Hospital of Guangzhou Medical University in Guangzhou, China, between January 1, 2009, and December 31, 2019. The patients were divided into two groups: HBsAg negative (36,500) and positive (3039). The viral load levels of 1250 HBsAg-positive women who had tested their HBV DNA load during pregnancy were separated into three groups. We utilized univariate and multivariable logistical regression analysis to determine the relationship between maternal chronic HBsAg carrier and GDM.

**Results:**

Being HBsAg positive was discovered to be an independent risk factor for GDM.Pre-pregnancy Obesity and advanced age were linked to an increased incidence of GDM. Those with a high HBV DNA load (> 10^6^ IU/mL) had a higher risk of GDM than HBsAg-positive women with a low viral load (< 10^3^ IU/mL). Pre-eclampsia and intrahepatic cholestasis of pregnancy (ICP) appeared to be more common in HBsAg-positive women than in uninfected women.

**Conclusions:**

Being HBsAg positive, advanced age, and pre-pregnancy obesity were all revealed to be independent risk factors for GDM in our study. In HBsAg carrier, pregnant women, a high HBV DNA burden was linked to a greater risk of GDM. Furthermore, being an HBsAg carrier during pregnancy raised the risk of ICP and pre-eclampsia.

## Introduction

Hepatitis B virus (HBV) infection is one of the most common global public health problems, with a high mortality rate, placing a heavy burden on health systems worldwide. The global prevalence of chronic hepatitis B surface antigen (HBsAg) varies widely, with rates ranging from 0.1 to 20% [1]. China is an intermediate endemic area for chronic HBV infection, with a rate of chronic HBsAg ranging from 5 to 6% [2–4]. It has been estimated that the prevalence of HBsAg among pregnant women in China varies from 6.3 to 9.4% [5–7]. Recent research has shown that carrying HBsAg during pregnancy increases the risk of gestational diabetes mellitus (GDM), but this result has not been found consistently [8–13]. Some studies have found that a high viral load is associated with a higher risk of GDM among pregnant women carrying HBsAg, but this result is inconclusive [11, 14]. GDM is a condition in which carbohydrate intolerance develops during pregnancy and is a common pregnancy complication. It has been estimated that a type of diabetes complicated 7% of pregnancies and that 86% of these women developed GDM [15]. When blood glucose levels were inadequately controlled, pregnant women with GDM increased pre-eclampsia risk and type II diabetes later in life. The offspring of pregnant women with GDM have a higher risk of developing macrosomia, neonatal hypoglycemia, and shoulder dystocia [16]. Considering many pregnant women suffer from HBV infection and GDM is related to a considerably higher risk of adverse maternal and neonatal outcomes, we completed a retrospective cohort study to explore the association of HBsAg with GDM to determine whether a high HBV DNA load is associated with a higher risk of GDM among maternal HBsAg carriers.

## Methods

### Database and study participants

We initially enrolled a total of 59,634 pregnant women from Guangzhou Maternal–Fetal Care Database [17] who gave birth between January 1, 2009 and December 31, 2019 at the Third Affiliated Hospital of Guangzhou Medical University. This database includes extensive information on hospital births collated from the electronic medical record system. The records were coded by qualified medical record staff using standardized definitions [18]. The database also contains information on maternal and neonatal demographic characteristics (age, history of pregnancy, gestational age, pre-pregnancy weight, pre-pregnancy body-mass index (BMI), smoking, and alcohol drinking during pregnancy, history of polycystic ovary syndrome (PCOS), birth weight, Apgar score, those who delivered male infants) and pregnancy outcomes(gestational hypertension, pre-eclampsia, GDM, intrahepatic cholestasis of pregnancy (ICP), pre-labor rupture of membranes (PROM), polyhydramnios, oligohydramnios, and postpartum hemorrhage (PPH), preterm birth, fetal distress, asphyxia conjunctivitis, macrosomia, fetal growth restriction (FGR) or transfer to the neonatal intensive care unit (NICU)). Medical conditions among mothers and neonates were coded using the International Statistical Classification of Diseases and Related Health Problems, 10th Revision (ICD-10, WHO). All individuals’ pre-pregnancy weight and height were measured, obtained from their healthcare records or self-reported, and BMI was calculated by multiplying their weight (kg) by their height squared (m^2^). In antepartum examinations, trained medical record personnel interviewed and recorded whether pregnant women smoked and drank alcohol during pregnancy.

We excluded 6398 participants who did not undergo a pre-pregnancy HBV serological test, 2677 women with chronic conditions (including hypertension, pre-existing diabetes mellitus, cardiac disease, and respiratory disease), and 1109 participants with other hepatitis viruses, human immunodeficiency virus, and/or treponema pallidum. We also excluded 3773 participants who had abortions; 2917 with multiple births; and 3221 women with missing data. The remaining 39,539 participants were used for the study.

**Fig. 1:**
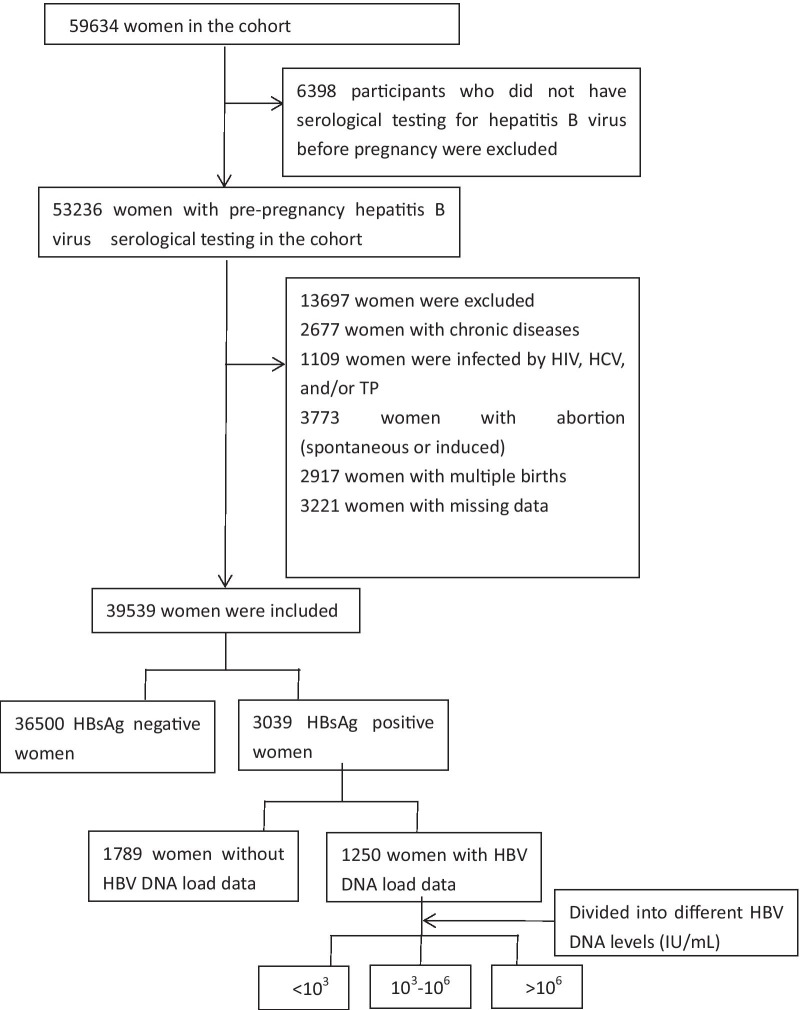
Profile of Study participants selection

Study participants were divided into two groups based on their HBsAg carrier status before pregnancy. The HBsAg negative group included 36,500 non-HBsAg carriers, and the HBsAg positive group included 3039 women with a history of HBV infection for more than 6 months, coupled with normal alanine aminotransferase (ALT) levels. We measured the HBV DNA load of 3039 HBsAg-positive women in the second trimester of pregnancy. We excluded 1789 HBsAg-positive women without viral load data. The remaining 1250 HBsAg positive women were divided into three groups based on their HBV DNA load levels: < 10^3^ IU/mL, 10^3^–10^6^ IU/mL, and > 10^6^ IU/mL (Fig. 1). This study was conducted per the requirements of the Declaration of Helsinki. All participants provided informed consent, and the appropriate ethics review board approved the study design.

### Outcomes

The major outcome of our study is the existence of GDM. The diagnostic criteria for GDM were defined as an oral glucose tolerance test (OGTT) during 24–28 weeks of gestation yielding one or more of the following results: a fasting plasma glucose level > 5.1 mM/L, a 1-h plasma glucose level > 10 mM/L, and/or a 2-h plasma glucose level > 8.5 mM/L [19].

### Data analysis

In univariate analyses, continuous variables were expressed as the mean ± standard deviation and tested using *t*-tests. Categorical data were compared by chi-square tests. We used univariate and multivariable logistical regression analysis to define the relation between maternal chronic HBsAg carrier and GDM and reported odds ratios (OR) and 95% confidence intervals (CIs). We carried out all analyses on SPSS 19.0, with a significant level at *P* < *0.05*.

## Results

Of the 39,539 women enrolled in the study, 3039 (7.7%) women tested positive for HBsAg. The average age of HBsAg positive women was 30.23 ± 4.60 years, which was older than the HBsAg negative women (29.53 ± 4.38, *P* < 0.05). Compared with the HBsAg negative women, those infected with HBV were more likely to be over 35 years, but not significantly so (18.6% vs. 13.8%, *P* > 0.05). HBsAg positive women had a greater gravidity history (2.08 ± 1.25 vs. 1.82 ± 1.07, *P* < 0.05) and parity history (0.50 ± 0.61 vs. 0.36 ± 0.51, *P* < 0.05) than HBsAg negative women. More women were categorized as normal weight in the HBsAg positive group than in the HBsAg negative group (76.4% vs. 61.7%, *P* < 0.05). The rate of male infant birth was 58.4% for HBsAg carriers and 45.8% for non-HBsAg carriers (*P* < 0.05). No statistical differences were present in the other maternal and infant characteristics between the two groups (Table 1).

**Table 1:** Maternal and neonatal characteristics for HBsAg status

	HBsAg positive (*N* = 3039)	HBsAg negative (*N* = 36,500)	*P* value
*Maternal characteristics*			
Age (years)	30.23 ± 4.60	29.53 ± 4.38	< 0.05
< 35	2473 (81.4%)	31,460 (86.2%)	< 0.05
≥ 35	566 (18.6%)	5040 (13.8%)	0.12
Gravidity	2.08 ± 1.25	1.82 ± 1.07	< 0.05
Parity	0.50 ± 0.61	0.36 ± 0.51	< 0.05
Multipara	1695 (55.8%)	24,032 (65.8%)	0.24
Gestational age (weeks)	38.27 ± 2.19	38.55 ± 2.17	< 0.05
Pre-pregnancy weight (kg)	56.70 ± 8.91	56.33 ± 8.76	0.76
Pre-pregnancy body mass index (kg/m^2^)	22.31 ± 3.32	22.17 ± 3.55	0.56
Underweight (< 18.5)	168 (5.5%)	3725 (10.2%)	0.97
Normal weight (18.5 ~ 23.9)	2323 (76.4%)	22,517 (61.7%)	< 0.05
Overweight (24 ~ 27.9)	386 (12.7%)	8383 (23.0%)	0.10
Obesity (≥ 28)	162 (5.4%)	1875 (5.1%)	0.54
Smoking during pregnancy	21 (0.7%)	219 (0.6%)	0.75
Alcohol drinking during pregnancy	36 (1.2%)	547 (1.5%)	0.81
*Neonatal characteristics*			
Birthweight (g)	3086.50 ± 557.18	3122.96 ± 531.07	0.06
Apgar score (1 min)	9.66 ± 1.25	9.70 ± 1.23	0.41
Apgar score (5 min)	9.84 ± 1.08	9.85 ± 1.08	0.25
Apgar score (10 min)	9.88 ± 1.03	9.87 ± 1.07	0.63
Male infants	1773 (58.3%)	16,712 (45.8%)	< 0.05

**Fig. 2:**
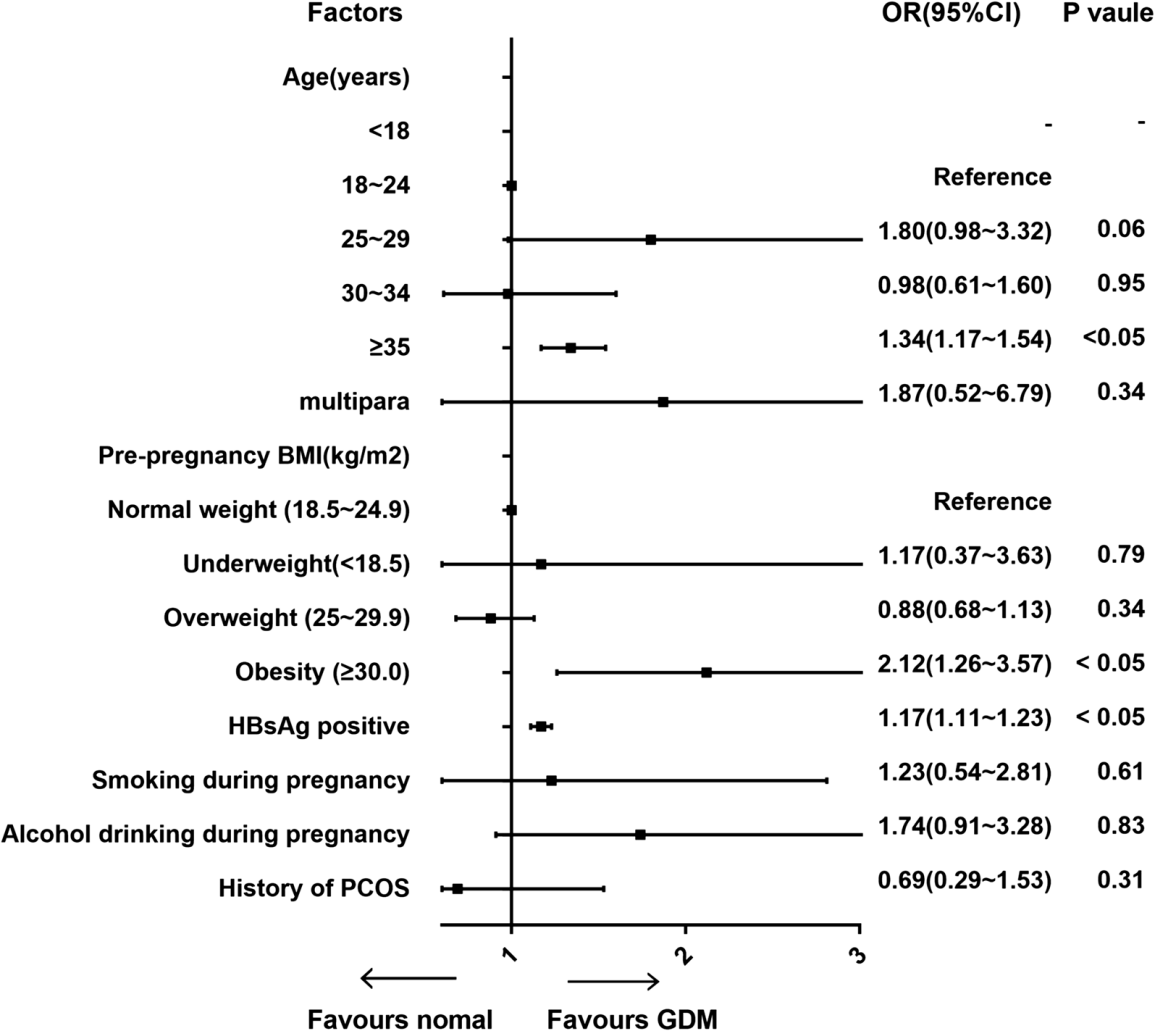
Univariate logistic regression analysis of factors related to GDM

**Fig. 3:**
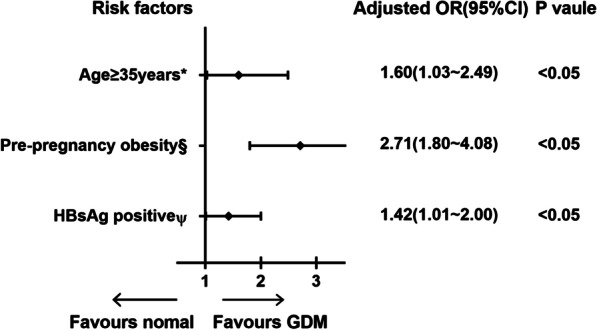
Multivariate logistic regression analysis of risk factors related to GDM. *Adjusted for gravidity and parity, gestational age, pre-pregnancy BMI, HBsAg carrier status. § Adjusted for age, gravidity and parity, gestational age, HBsAg carrier status. Ψ Adjusted for age, gravidity and parity, gestational age, pre-pregnancy BMI

Our study found that HBsAg-positive women had an increased risk of GDM compared to those without an HBV infection (12% vs. 9.7%, *P* < 0.05). HBsAg positive women appeared to have a higher risk of pre-eclampsia (3.4% vs 2.5%, *P* < 0.05) and ICP (1.1% vs. 0.2%, *P* < 0.05) than HBsAg negative women. Other pregnancy complications and neonatal outcomes had no significant discrepancy between 2 groups (Table 2). To define whether being a chronic maternal HBsAg carrier was an independent risk factor of GDM, univariate and multivariable logistical regression analyses were performed on the related risk factors of GDM, including age, multipara, pre-pregnancy BMI, HBsAg positive, smoking, and alcohol drinking during pregnancy and any history of PCOS. In the univariate logistic regression analyses, being HBsAg positive and aged ≥ 35 years were associated with a higher risk of GDM, with OR value of 1.17 (95% CI 1.11–1.23) and 1.34 (95% CI 1.17–1.54), respectively. Compared with normal-weight women, pre-pregnancy obesity was related to an increased risk of GDM (OR 2.12, 95% CI 1.26–3.57). However, there was no significant association between GDM and other factors, including multipara, smoking and alcohol drinking during pregnancy, and PCOS history (Fig. 2). The multivariable logistic regression analyses found that being HBsAg positive was an independent risk factor of GDM (OR 1.42, 95% CI 1.01–2.00). Furthermore, advanced age and pre-pregnancy obesity were independent risks of GDM after adjustment for other variables (Fig. 3).

**Table 2:** Maternal and neonatal outcomes for HBsAg status

	HBsAg positive (*N* = 3039)	HBsAg negative (*N* = 36,500)	*P* value
*Maternal outcomes [N (%)]*			
Gestational hypertension	18 (0.6%)	242 (0.7%)	0.64
Pre-eclampsia	103 (3.4%)	924 (2.5%)	< 0.05
GDM	366 (12.0%)	3529 (9.7%)	< 0.05
ICP	32 (1.1%)	90 (0.2%)	< 0.05
PROM	213 (7.0%)	2763 (7.6%)	0.26
Oligohydramnios	265 (8.7%)	3378 (9.3%)	0.32
Polyhydramnios	14 (0.5%)	158 (0.4%)	0.82
PPH	152 (5.0%)	2159 (5.9%)	0.05
*Neonatal outcomes [N (%)]*			
Preterm birth	119 (3.9%)	1720 (4.4%)	0.22
Fetal distress	149 (4.9%)	1728 (4.7%)	0.67
Asphyxia neonatorum	52 (1.7%)	496 (1.4%)	0.11
Overweight offspring	4 (0.1%)	1029 (2.8%)	0.57
FGR	64 (2.1%)	790 (2.2%)	0.83
Transfer to the NICU	434 (14.3%)	4417 (12.1%)	0.35

**Fig. 4:**
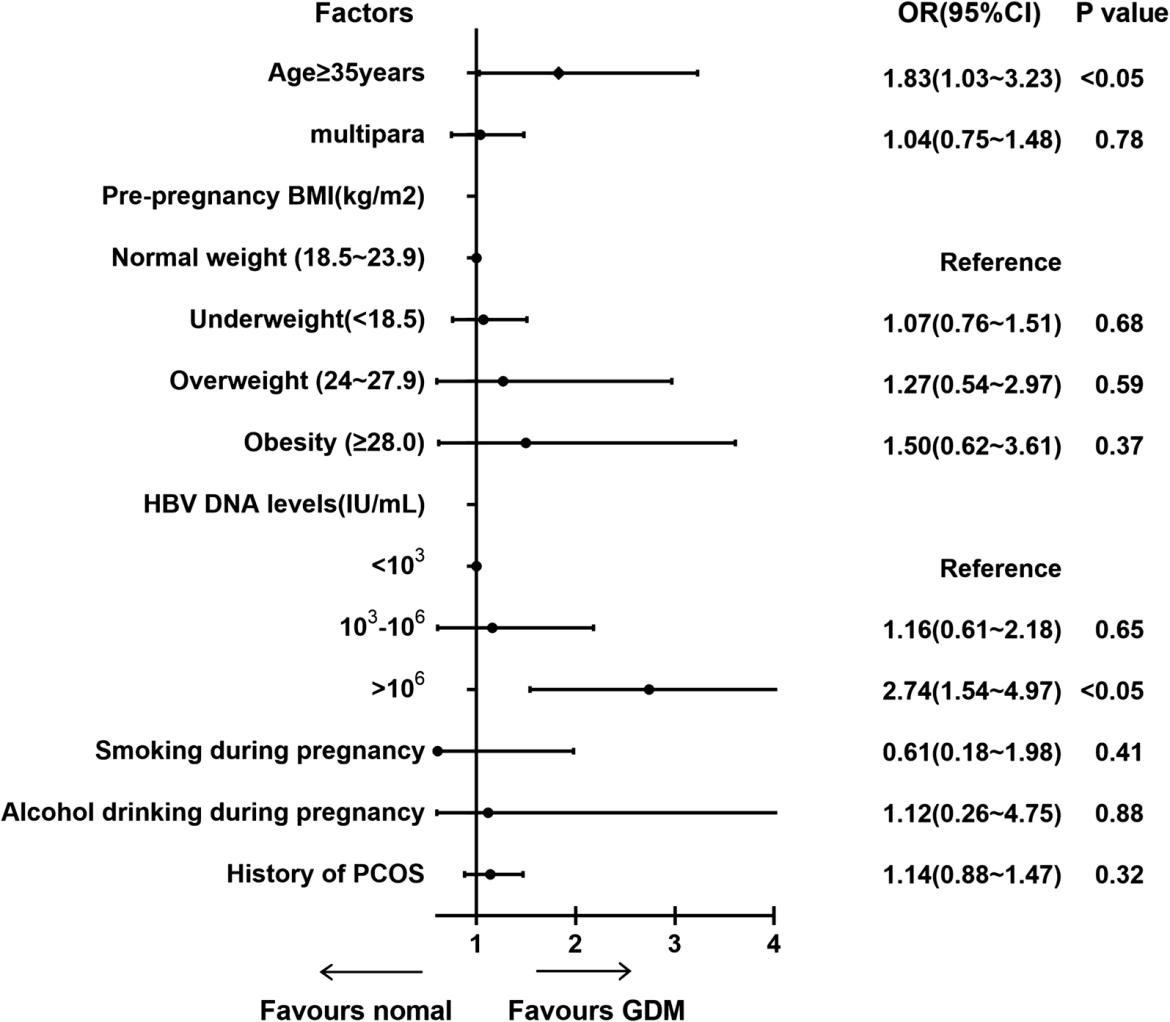
Univariate logistic regression analysis of factors related to GDM in positive HBsAg women

**Fig. 5:**
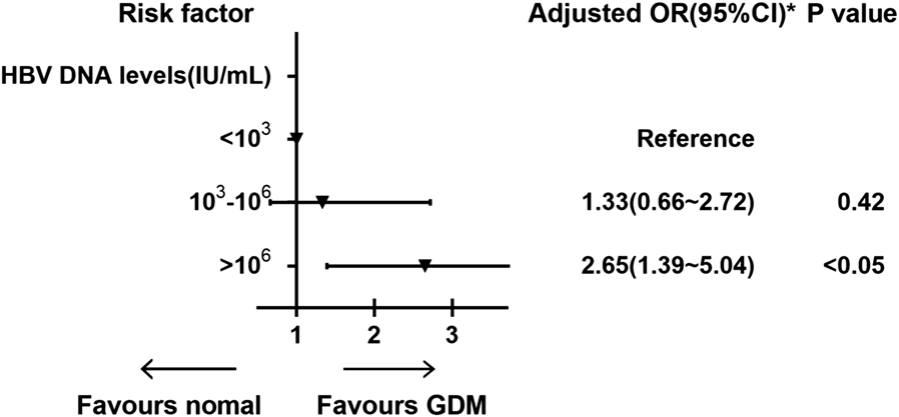
Multivariate logistic regression analyses of risk factor related to GDM in HBsAg positive women. *Adjusted for age, pre-pregnancy BMI

We performed further analyses to determine whether a high HBV DNA load was associated with a higher risk of GDM among HBsAg-positive women. In total, 1250 HBsAg positive women who had the tested HBV DNA load during pregnancy were divided into three groups based on viral load levels: low viral load (< 10^3^ IU/mL), medium viral load (10^3^–10^6^ IU/mL), and high viral > 10^6^ IU/mL. Compared with HBsAg-positive women with low viral load(< 10^3^ IU/mL) and medium viral load(10^3^–10^6^ IU/mL), those with high HBV DNA load (> 10^6^ IU/mL) had a higher incidence of GDM (Table3). Univariate and multivariable logistical regression analyses were performed to determine whether a positive relationship exists between increased HBV DNA load and elevated risk of GDM or a dose–effect relationship. In the univariate logistic regression analyses, compared to HBsAg positive women who had a low HBV DNA load (< 10^3^ IU/mL), those who had a high viral load (> 10^6^ IU/mL) were associated with a higher risk of GDM (OR 2.74, 95% CI 1.54–4.97). Aged ≥ 35 years was associated with an increased risk of GDM in HBsAg-positive women (OR 1.83, 95% CI 1.03–3.23) (Fig. 4). In the multivariable logistic regression analyses, we found that high HBV DNA load was an independent risk factor of GDM among HBsAg positive women, with OR values of 2.65 (95% CI 1.39–5.04) (Fig. 5).

**Table 3:** Maternal characteristics for HBV DNA levels of HBsAg positive women

	< 10^3^ IU/mL (*n* = 653)	10^3^ − 10^6^ IU/mL (*n* = 315)	> 10^6^ IU/mL (*n* = 282)	*P* value
Age (years)	28.23 ± 3.59	26.93 ± 3.38	29.14 ± 4.11	< 0.05
< 35	551 (80.5%)	277 (87.9%)	232 (82.1%)	0.82
≥ 35	102 (15.6%)	38 (12.1%)	50 (17.9%)	< 0.05
Gravidity	1.68 ± 1.31	1.32 ± 1.12	1.17 ± 1.69	0.42
Parity	0.30 ± 0.78	0.39 ± 0.57	0.60 ± 0.45	0.07
Pre-pregnancy body mass index (kg/m^2^)	21.24 ± 4.02	22.35 ± 1.58	23.01 ± 1.59	0.05
Underweight (< 18.5)	221 (8.7%)	3725 (10.2%)	28 (9.8%)	0.59
Normal weight (18.5–23.9)	2128 (65.3%)	22,078 (60.5%)	167 (59.4%)	0.13
Overweight (24–27.9)	528 (18.1%)	8383 (23.0%)	64 (22.6%)	< 0.05
Obesity (≥ 28)	162 (7.9%)	2314 (6.3%)	23 (8.2%)	0.71
GDM	48 (7.4%)	55 (9.5%)	53 (11.6%)	< 0.05

## Discussion

Most pregnant women with HBV infection were classified as chronic HBsAg carriers [12]. The global prevalence of HBsAg in pregnant women has been estimated to be 5.0% [20]. In China, the prevalence of maternal HBsAg has declined in recent years; however, it was higher than the global average [21]. According to a study from 2018, the prevalence of HBsAg in pregnant women has declined from 9.8 to 6.3%, while more recent studies have reported a prevalence ranging from 7.0 to 9.4% [5–7]. In our retrospective cohort of 39,539 women, we found that 7.7% of pregnant women tested positive for HBsAg, consistent with the latter studies.

Recent reports have found that being a chronic HBV carrier was associated with adverse pregnancy outcomes such as GDM [22–24]. GDM was traditionally defined as carbohydrate intolerance of variable severity with onset or first detection during pregnancy. The latest meta-analysis from 2019 showed that 14.8% of pregnancies in China were complicated by GDM, increasing prevalence [25]. GDM was one of the most common medical complications during pregnancy, associated with adverse pregnancy outcomes, including hypertensive disorders, preterm labor, neonatal hypoglycemia, hyperbilirubinemia, and respiratory distress syndrome. It was necessary to highlight the significance of understanding risk factors and specifically modifiable factors for GDM and prevent the condition, which might lower the risk of GDM-related adverse maternal and neonatal outcomes. Nowadays, the most common risk factors of GDM include older maternal age, obesity, and a family history of diabetes [26]. Whether being HBsAg positive, a history of PCOS, and smoking or alcohol consumption during pregnancy are risk factors of GDM remains uncertain.

In our retrospective cohort of 39,539 women, we found that maternal HBsAg carriers have a higher risk of GDM (12% vs. 9.7%, OR 1.42, 95% CI 1.01–2.00). Being HBsAg positive was an independent risk factor of GDM. Several studies supported this finding. Lao et al. [8] analyzed 13,683 women in Hong Kong between 1998 and 2001 and found that the rate of GDM was statistically higher in pregnancies with an HBV infection (12.4 vs. 10.2%, OR 1.24, 95% CI 1.01–1.51). In 2016, Tan et al. [10] concluded that the rate of GDM in HBsAg carriers was higher than non-HBsAg carriers (11.8 vs. 8.2%, OR 1.41, 95% CI 1.15–1.74) in a case–control study 22,374 Chinese women. The latest meta-analysis from 2018 demonstrated that women with HBV infection had an increased risk of GDM (6.48% vs. 3.41%, OR 1.35, 95% CI 1.17–1.56) [11]. However, a few previous studies have shown the connection between maternal HBV infection and the development of GDM to be insignificant. Reddick et al. [12] showed that the incidence of GDM among HBsAg positive women (4.4%) was similar to that of HBsAg negative women (2.5%, *P* > *0.05*). Cui et al. [13] found the incidence of GDM between the HBV infected women and non-HBV women to be insignificant (1.17% vs. 1.13%, *P* > 0.05) in a prospective cohort study involving 21,004 pregnant women. Furthermore, our study found that older maternal age and obesity were associated with a higher risk of GDM, consistent with the previous studies.

The potential mechanism for the correlation between HBsAg and GDM is unclear. The liver is an important organ that handles liver glucose metabolism and the development of insulin resistance. Liver disease and inflammation caused by HBV during pregnancy may lead to reduced insulin sensitivity [27]. Another mechanism could be that HBV-infected pregnant women display a spontaneously increased production of tumor necrosis factor-alpha, which contributes to insulin resistance leading to the development of GDM [28, 29].

Additionally, we found that a high HBV DNA load (> 10^6^ IU/mL) was an independent risk factor of GDM among HBsAg-positive pregnant women. A recent meta-analysis supported our finding. The latter study found that HBsAg positive had a 47% higher risk of developing GDM than HBV negative women, associated with HBV serological markers, including HBV DNA load [11]. However, Peng et al. found that HBV infection during pregnancy was an independent risk factor for GDM, but the phenomenon is not related to viral activity such as hepatitis B e-antigen status and viral load [14]. This phenomenon might be because HBV DNA load reflected the level of virus replication and infectivity in patients with an HBV infection. There was a strong inflammatory response in HBV-infected women with a high HBV DNA load, leading to a higher risk of GDM. Furthermore, our study showed that an HBV infection during pregnancy increases the risk of ICP (1.1% vs. 0.2%, *P* < 0.05) and pre-eclampsia (3.4% vs. 2.5%, *P* < 0.05). These findings, which have rarely been investigated before, may have important clinical implications for pregnant women [30, 31].

Given that maternal HBsAg was associated with an increased risk of adverse pregnancy outcomes, our study suggests that women should receive a serological test for HBV infection before or early in the pregnancy. Thus, it is necessary to establish appropriate diagnosis and management for HBV-infected women. We recommend that HBV DNA load and the liver function of HBsAg-positive women be monitored regularly, which might help observe any change of condition. Antiviral treatment might be considered for women with a high HBV DNA load when necessary [32]. Moreover, the risk of adverse pregnancy outcomes in HBV-infected pregnant women should not be ignored. Screening and early interventions for GDM among high-risk populations such as those who are HBsAg positive (especially those with a high HBV DNA load), are older at maternal age, and are obese might contribute to the prevention or early diagnosis of GDM [33].

The strengths of the current study included a large sample size and comprehensive information on demographic characteristics, maternal and neonatal outcomes. However, our study was a single-center retrospective cohort study, which could not exclude selection and information bias. Additionally, we did not investigate the physiological mechanisms of the association between HBsAg and GDM.

## Conclusion

Above all, our study found that being HBsAg positive, advanced age, and pre-pregnancy obesity were independent risk factors of GDM. High HBV DNA load (> 10^6^ IU/mL) was associated with a higher risk for GDM among HBsAg-positive pregnant women. In addition, being positive for HBsAg during pregnancy increased the risk of ICP and pre-eclampsia.

## Data Availability

The datasets used and/or analyzed during the current study are available from the corresponding author on reasonable request.

## Abbreviations

HBV: Hepatitis B virus
HBsAg: Hepatitis B surface antigen
GDM: Gestational diabetes mellitus
ALT: Alanine aminotransferase
OGTT: Oral glucose tolerance test
BMI: Body-mass index
ICP: Intrahepatic cholestasis of pregnancy
PROML: Pre-labor rupture of membranes
PPH: Postpartum hemorrhage
FGR: Fetal growth restriction
NICU: Neonatal intensive care unit
OR: Odds ratios
CIs: Confidence intervals
PCOS: Polycystic ovary syndrome
